# Study of Rh/TiO_2_–SiO_2_ system in photolytic water splitting

**DOI:** 10.1007/s11144-020-01924-3

**Published:** 2021-02-07

**Authors:** Jolanta Wasilewska, Michał Chmielarek, Wincenty Skupiński

**Affiliations:** 1 Organic Technology and Catalytic Division, Faculty of Chemistry, Warsaw University of Technology, Noakowskiego 3, 00-664 Warsaw, Poland; 2 High Energetic Materials Division, Faculty of Chemistry, Warsaw University of Technology, Noakowskiego 3, 00-664 Warsaw, Poland

**Keywords:** Rh/TiO_2_–SiO_2_ (TiO_2=_ 0–100 mol %), Photowater splitting reaction, Mechanism

## Abstract

Titania silicates (TiO_2_–SiO_2_) of various compositions were prepared by a sol–gel method. RhCl_3_ was used in 0.2 wt% of Rh quantity on the gel surfaces, which were subsequently exposed to UV irradiation in water under a 355-W UV lamp. λ > 370 nm. Both Rh [I] and Rh [III] surface complexes were formed, depending on the gels composition. They also exhibited various efficiency in photo water splitting reaction, the photocatalysts possessing as supports titania-silica gels containing 40 and 50 mol % of TiO_2_, were the most effective. According to our proposal, the rhodium complexes took part in the oxidative addition and reductive elimination cycle, where two water molecules in photo water splitting reaction, yielding hydrogen – H_2_ and two hydroxyl radicals. The two hydroxyls radicals, catalytically converted to oxygen atom and water, in the second cycle of the investigated water splitting reaction. Titanium octahedra and silicon tetrahedra linked by Ti–O–Si bridges were thought to be the grouping responsible for activity of the investigated catalysts. Their largest amounts are on the gels containing 40–58 mol % TiO_2._

## Introduction

Titanium dioxide is well known as photocatalyst active in many processes e.g.: the environmental and energy fields, including self-cleaning surfaces, air and water purification systems, sterilization, hydrogen evolution, and photoelectrochemical conversion [1–4] but also as materials for photoeletrodes applied in water electrolysis, which is the most effective in solar water splitting processes [5–9]:

Its activity in these processes, is enhanced while determined metal or their oxide are deposed on its surface [10–12].

A very expressive example of TiO_2_ photocatalysts activation by metals, the systems Ag–TIO_2_ possessing various amount of silver, may be [13].

It was applied to photo-decompose an OOL dye, under visible light. While the catalyst Ag, TiO_2_, decomposed the dye, used in experiment quantity, almost completely, the bare TiO_2_ support decomposed only half.

Also such an effect can be reached while silicon atoms are incorporated to the titanium oxide lattice, while titania-silica and TiO_2_ were applied as photocatalysts in the water splitting reaction. The titania –silica containing 50 wt% could produce 25 μmol of hydrogen/24 h, while only traces of hydrogen were determined, for titania, under the same experimental conditions [14]

In other work, when titania-silica gels of various compositions were investigated, the highest acidity and connected with it the highest efficiency in hydrogen oxide decomposition, were observed when the gel possessed 50 mol % of TiO_2_ [15]

Deposition of metals on these mixed gels as: Rh, Ni, Cr, Co, V, or their oxides yields effective catalysts in such reactions as benzene and CO hydrogenation, ethylene polymerization, reduction of CO by means of NO, aldehyde condensation or oxidation of aromatic compounds [16].

Titania-silica gels (TiO_2_–SiO_2_), obtained by wet hydrolysis of various hydrolysable compounds of titanium and silicon, feature a wide variety of physicochemical and structural properties, that depend on their composition, i.e. on the titanium and silicon content [14–19]. In all these gels Ti–O–Si bond occurs which bring in new, active, specific centers of an activity that exceeds the centers on the silica and titania gels alone.

However, electronegativity of these elements differ. For titanium it is 1.54, for silicon, 1.90 [16]. Thus the silicon ion will be more electron-accepting in this bond which results in a shift of the electron cloud from titanium onto the oxygen ion and further on to silicon ion. This effect will be favored by an increased lability of titanium ion electrons than of silicon ion, as in the titanium ion the 3p electrons are more distant from the nucleus than the 2p electrons in the silica ion (it will be more strongly attracted by the positively charged nucleus).

This will cause the titanium ion acquire properties of a relatively strong Lewis acid, the oxygen ion so the character of relatively strong Lewis base, whereas the silicon ion Lewis- acid properties will be reduced.

This acceptor effect depends on in what molecular structure the titanium ion will be in these mixed gels.

If the molar content of TiO_2_ in these gels does not exceed 10% the co-ordination number of the major amount of titanium ion equals four and only a minor amount of titanium ions ca. 10% have a co-ordination number six.

In tetrahedral structures oxygen ions are exposed on surfaces and in the case of silicate tetrahedral surface hydroxyl groups -SiO_3_(OH)˥^4−^ and in smaller amount of -TiO_3_(OH)˥^4−^ may occur. This ensures easier access of the particles interacting with them. The both hydroxyls posess specific IR bands: 3743 cm^−1^ (isolated Si–OH)) and 3615 cm^−1^ (Ti–OH) [16], but after the gels annealing at temperature higher than 773 K, only silanol groups remain.

If the TiO_2_ content in gels is higher than 10 mol %, the quantity of octahedral titanium structures increases at the expense of the tetrahedral ones. The amount of the titanium octahedron-silica tetrahedron structures will predominate up to 50 mol % TiO_2_ gels.

Also, structures with titanium ions of the co-ordination number five emerge. These are certainly located in surface beheaded octahedral and oxygen vacancies [12]. Also in this range of gel composition the Ti–O–Si grouping occur which bind titanium tetrahedra and octahedra with silicon tetrahedral.

After calcinations of these gels at temperatures higher than 773 K and their TiO_2_ content rises above 50 mol %, there appears the titanium oxide phase of anatase and rutile structures occur, about dimensions of 8–23 nm, what destroys these gels lattice and dramatic decreases their specific areas [16, 18].

These data indicate that every composition of titania-silica gels will have its specific properties which may translate into their diversified physicochemical properties and applications.

The gels under consideration exhibit various acidity that primarily depends on composition and the annealing temperature during the preparation process [20]. The acidity is examined by a variety of techniques, mostly by the adsorption of basic indicators technique [15, 19]. The recording of IR spectra of these adsorbed indicators, through measurements of the intensity and shifts of the positions of the suitable bands in the spectrum, allows to determine the amount and acid strength of the centers on surface of the gels under investigation.

The methods employed allowed to determine the amount of the acidic centers of the Brønsted and of the Lewis types. The quantity of these centers depends on the method of preparation of the gels under study and on the temperature of their calcinations [19]. Either of these centers were present in the highest number on the gels prepared by co-precipitation technique calcined at 350 °C and containing 44 to 92 mol % titanium. The catalysts were tested in the reaction of dehydration of methanol to diethyl ether. In this reaction a relatively high activity was observed in the titanium oxide itself which possesses merely the Lewis-type acidic centers. However, the mixed-type titania-silica gels of the composition mentioned, with less Lewis-acid centers, but also with Brønsted acid centers, were more active. These gels were amorphous.

A rise of calcination temperature to 550 °C resulted in a decrease in the amount of both acidic centers in the catalysts under study and in a drop in their activity.

Titania-silica gels, as mentioned above, are also active in the photolytic reaction of decomposition of water to hydrogen and oxygen. Study on the catalyst of a composition 50 wt% TiO_2_ −50 wt% SiO_2_ (36 mol % TiO_2_–64 mol % SiO_2_), showed a higher effectiveness in this reaction than bare TiO_2_ [14].

The study made on this mixed gel, concerned with the following properties: band-gap energy, flat band potential, and doping density showed that the photocurrent density of TiO_2_–SiO_2_ mixed oxide is lower than that of bare TiO_2_ and the photocurrent density of TiO_2_–SiO_2_ mixed oxide is lower than that of bare TiO_2_. The data point to the excellent effectiveness of TiO_2_–SiO_2_, system in this photolytic reaction, to the higher flat band potential, band-gap energy, and doping density than those of bare TiO_2_.

It has been postulated that the Ti–O–Si groupings are responsible for the high catalytic activity of these mixed gels. It should be also mentioned that the bridges will differ due to diversified environments, namely the SiO_4_ tetrahedron and the TiO_6_ octahedron. [16]

The various structures of titania-silica gels are likely to ensure, through specific interactions, various physico-chemical and catalytic properties of metal ions deposited on their surfaces [16].

When examining the system, namely rhodium ions deposited on titania-silica gels of various compositions: 0 ÷ 100 mol % TiO_2_, we wanted to determine how these gels would affect the physico-chemical and catalytic properties of rhodium ions deposited on them as well as their interaction in the photolytic water splitting reaction under investigation.

The results of these studies and the proposals of conversions taking place during the investigated reaction are presented in the paper below.

## Experimental section

### Materials

In the preparation procedure for titania-silica gels the following reagents were used: aqueous ammonia 25% analytical purity grade (cz.d.a.) POCH Gliwice (Poland); Isopropyl alcohol cz.d.a., POCH Gliwice (Poland); Tetraethoxysilane, Sigma Aldrich, purified by distillation; Tetraethoxytitanium, 97% purity, Sigma Aldrich. For the preparation of rhodium catalysts, rhodium (III) chloride hydrate (Rh, 38–40%) (Sigma Aldrich) was used.

### Preparation of titania-silica gels

Titania-silica gels were made, according to the literature data [14, 15] by wet hydrolysis of isopropanol solution of tetraethoxysilane and tetraethoxy titanate using 25% ammonia water as a precipitant in a suitable quantity of distilled water. Quantities of suitable amounts of these reagents to prepare theoretical compositions of particular gels are collected in Table 1.

**Table 1:** The amounts of tetraethoxy titanate and tetraethoxysilane required to make theoretical compositions of the titania-silica gels examined

TiO_2_/SiO_2_	Mol %	0/100	10/90	20/80	30/70	40/60	50/50	60/40	70/30	80/20	90/10	100/0
(C_2_H_5_)_4_Ti	G	0	2.9	5.7	8.6	11.4	14.3	17.1	20.0	22.8	25.7	28.5
(C_2_H_5_)_4_Si	G	34.6	23.4	20.8	18.2	15.6	13.0	10.4	7.8	5.2	2.6	0

### Example description of the preparation of the 50 mol % TiO_2_ – 50 mol % SiO_2_

In a round-bottomed flask of a 500 cm^3^ capacity titanium tetraethoxide (14.74 g) and silicon tetraethoxide (13.00 g) were dissolved in 200 cm^3^ of isopropyl alcohol. The flask contents under a vigorous stirring was heated on a heating bowl until boiling. Then 7.8 cm^3^ ammonia water was added into the solution that made a white precipitate to form. Addition of a small amount of ammonia not to exceed 1 wt% suffices to make silicon hydroxide to precipitate completely.

The suspension thus obtained was vigorously stirred under a gentle boiling for one hour. Upon cooling of the reaction flask the precipitate was filtered off under a reduced pressure using a water-suction pump. Next, the product was washed several times with distilled water to rinse off the remainder of isopropanol and HCl (Ag test). The wet precipitate was fried successively for 24 h at three temperatures, i.e.: 333 K, 373 K and 423 K. Upon comminution of the product on sieves down to a < 0.04 cm fraction it was placed in a quartz vessel.

The titania-silica gel obtained was subjected to a calcining process for 24 h at 823 K. Upon concluding all the processing stages the ready gel was stored in a desiccator with potassium hydroxide as a drying agent. Their specific areas were determined applying the BET method.

### Photocatalyst preparation [21]

Solution (9.5 mM) of RhCl_3_ in 30 cm^3^ distilled water was prepared. In a Schlenk-type quartz round-bottomed flask (50 ml capacity) were placed: 1.08 ml of prepared rhodium chloride solution and 0.55 g of the carrier. In an (air-conditioned), blackened, fume hood a flask was placed that was filled with a magnetically stirred suspension. The system was outgassed for 15 min under a pressure of 15 Torr. Subsequently, the system was exposed to the light from a POLAMP PLK-85 of a power rating of 355 W quartz lamp that emitted UV–VIS radiation for 8 h at a temperature of ca. 323 K. The purpose of the light treatment was to make the rhodium settle from the solution on the carrier surface.

Upon catalyst photoactivation procedure the contents of the quartz flask was transferred to an evaporator. In the evaporator the whole water contained in the suspension was evaporated under a reduced pressure of 15 Torr. Thus, the photocatalyst ready for use in water photolysis was obtained.

The degrees of oxidation of so deposited rhodium compounds were determined by the ESCA method using ESCA VG ESCA-3 apparatus (internal reference standard Ti 2p_3/2_ = 459.0 eV).

### Water photolysis reaction standard conditions

Photoactivated catalyst (0.05 g) (0.2% Rh) was placed in a Schlenk quartz flask of a capacity of 50 ml and was poured with 5.5 cm^3^ of re-distilled water maintaining a pH   7. In the flask a magnetic stirring element was placed and the system was degassed under vacuum of 15 Torr (15 min). The setup was placed in a ventilated, darkened fume hood. The water photolysis process was conducted for 24 h under stirring of the photocatalyst water suspension illuminating the system with a quartz lamp POLAMP PLK-85 UV–VIS of a power rating of 355 W, at 326 K.

The amount of the hydrogen evolved was determined by means of GLC (cathetometer, 20 cm column, 3X screens 1, room temperature, helium as carrier gas.

## Results

The specific surfaces of titania-silica gels obtained, their actual composition (by the ESCA method -Si2p signal intensity) and theoretical excess of the SiO_2_ and TiO_2_ phases are presented in Table 2

**Table 2:** Physico-chemical properties of titania-silica gels obtained

TiO_2_/SiO_2_	mol%	0/100	10/90	20/80	30/70	40/60	50/50	60/40	70/30	80/20	90/10	100/0
Phase excess^a^	mol%	100_SiO2_	80_SiO2_	60_SiO2_	40_SiO2_	20_SiO2_	0	20_TiO2_	40_TiO2_	60_TiO2_	80_TiO2_	100_TiO2_
Si 2p (ESCA)	mol%	100	94	83	73	62	52	37	–	16	–	0
Specific area	m^2^/g	390	281	266	251	230	196	38	48	75	82	11

^a^Theoretically

In order to confirm the composition of titania-silica gels obtained after their preparation, ESCA analysis as carried out as regards the quantity of silicon atoms in gel samples. The results of examination, presented in Table 2, indicate the excess silicon atoms from 2 to 6% in gels 10–50 Ti and lower quantity of these atoms for gels 60–90Ti ranging from 2 to 8%. With this in mind, a decimal description of the symbols of the composition of gels will follow in the study.

The results of the ESCA analysis demonstrate that the calculated SiO_2_ and TiO_2_ phase excess values are also corresponding to their actual quantity in gels.

In titania-silica gels Ti–O–Si bonds are encountered, whereas Si–O–Si and Ti–O–Ti bonds appear only in appropriate excess phases. Taking this into consideration, Table 2 presents only excess phases depending on the composition of the gels obtained.

In a gel containing 10 mol % of TiO_2_ (for the sake of simplicity, the individual compositions of investigated gels will be referred to as, for instance: 10% TiO_2_ mol–90% SiO_2_ mol, as 10% Ti, and the percentage values will always refer to molar percentages. Titanium ions of this oxide will form Ti–O–Si bonds-the titanium-silica phase and free Si–O bonds in the silicate phase will theoretically Si bonds in the silicate phase will theoretically amount to 80 mol %. Excess of silicate phases decreases to zero for a 50% Ti gel.

When the TiO_2_ content increases to 60 mol%, the gel will still contain 40% of the titanium silica phase and 20% of the titanium oxide phase. While continuing to increase the amount of TiO_2_ in gels, the amount of TiO_2_ phase will increase to 80% for 90% TiO_2_ gel, which still contains 10% of the titanium silica phase.

These calculations point to the fact that in titanium silica catalysts containing relatively high excess of SiO_2_ or TiO_2_, these phases can significantly affect the physico-chemical and catalytic properties of these catalysts.

The analysis of the surface of titania-silica gels obtained using the BET method revealed that the silica gel exhibited the largest specific surface area, namely 390 m^2^/g; for 10% TiO_2_ gel this surface is much smaller and amounts to 281 m^2^/g. In other gels: 20–50% TiO_2_, this area gradually decreases, from 266 to 196 m^2^/g with increased amount of titanium ions. The introduction of more Ti ions entails a significant change to the surface of their grains. These changes are not continuous: the 60% TiO_2_ gel exhibited the surface area equal to 38 m^2^/g, and the 90% TiO_2_ carrier: 82 m^2^/g. TiO_2_ alone was characterized by the smallest specific surface area = 11 m^2^/g.

These data indicate that the formation of Ti–O–Si bonds is accompanied by a proportional change in the specific surfaces of mixed oxides under investigation, within the range of 0–50Ti. According to model tests, these bonds connect the layers of chains of the phases [20]. As the surfaces of micropores share the greatest contribution to the overall surface of inorganic oxide grains, e.g.: 3 specific surfaces of microporous inorganic oxides amount to 1000 m^2^/g, and for those macroporous oxides, the surfaces are contained in the range of 100–200 m^2^/g, whereas pore diameters: amount to 1–2 nm and over 50 nm [22]. Thus, in the excess SiO_2_ phases, Ti–O–Si bonds separate off the micropores that occur in the silica gel and entice a gradual decrease of specific surface area of 90-40Ti gels. Therefore, Ti–O–Si bonds are likely to block the entry to these pores.

50% Ti gel consists only of the titanium silica phase: TiO_2_ octahedron - SiO_2_ tetrahedron, with a specific surface area of 196 m^2^/g.

When a separate amorphous titanium oxide phase is present in titania-silica gels, then, during annealing above 773 K, as mentioned earlier, this phase is initially converted into the rutile crystalline form, and then into anatase crystals. This process is accompanied by the decrease of the surface area of such gels and the destruction of their surface forms [15, 23].

Crystals are usually characterized by relatively small specific surfaces and if they appear on the surfaces of investigated gels, they need to reduce their surface significantly, proportionally to the increased TiO_2_ phase content. However, in the case under study no proportionality between the amount of TiO_2_ in gels and the specific surface area was observed, namely the 60% TiO_2_ gel has a surface area of 38 m^2^/g, and 90% TiO_2_ gel: 84 m^2^/g.

In a 60% TiO_2_ gel, the phase composed of TiO_2_ octahedrons and SiO_2_ tetrahedra still occupies a significant area. The grains containing 50% TiO_2_ have a specific surface area of 196 m^2^/g and 60% TiO_2_ gel should be expected to have a slightly lower surface area. Presumably, the crystallization of 20% TiO_2_ to anatase proceeds with closed micropores of the titanium silica phase, possibly by blocking the entries to these pores with anatase crystals. Their dimensions extend from 8 to 23 nm [23], and the entries to the micropores amount to 2 nm. The nucleation and formation of crystals is also taking place at “hot spots” on solid surfaces, namely the edges of surface layers [24], for instance, pore entry points and the running anatase crystallization yields the described effect.

The relatively large specific surface of the 90% TiO_2_ gel probably is the result of the construction of a relatively large number of anatase crystals of large dimensions, only at the expense of the TiO_2_ phase itself and the exposure of the titanium silica phase.

The absence of this phase in TiO_2_ alone must have resulted in correspondingly large anatase crystals of specific surface area of 11 m^2^/g.

These effects work together in other gels in this range, producing diversified specific surface areas.

The acidity tests of the obtained titania-silica gels by Hammett indicators method revealed poor acidic properties of gels amounting to H_o=_  ≤  + 2.8 strength. It corresponds to the 0.002% of acid strength of sulfuric acid. This method does not distinguish between the character of Brønsted-type acid centers and Lewis-type acid centers.

Lewis-type acid surface catalytic centers may accept one or two electrons from donor compounds [25]. The application of a single-electron donor terylene adsorption in the investigation aimed to determine the actual character of acid centers of the gels under study. The results of these tests are presented in Table 3.

**Table 3:** Acidity and acceptor values of Ti-Si

TiO_2_/SiO_2_	Mol %	0/100	10/90	20/80	30/70	40/60	50/50	60/40	70/30	80/20	90/10	100/0
Acidity^a^	μ mol/g	0	0.08	0.15	0.30	0.50	0.45	0	0.02	0	0	0
Acceptor centers^b^	j.u	0	0.2	–	0.33	0.65	0.6	0.3	o.16	0.2	O2	0

^a^H_0_ ≤  + 2.8

^b^EPR perylene radicals

**Fig. 1:**
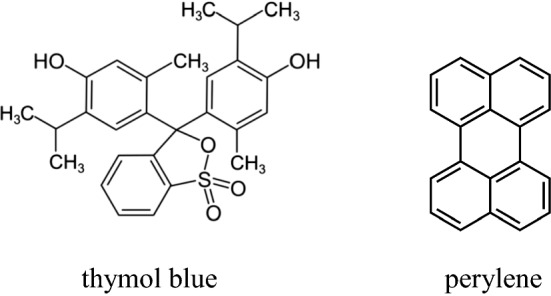
The structure of indicator used

The results obtained exhibit a corresponding quantity of both centers depending on the composition of investigated gels, within the range of 0 ÷ 50%TiO_2_, with a maximum value for 40–50% TiO_2_ for gels. For higher TiO_2_ content in gels, the centers of H_o_ ≤  + 2.8 strength are absent, however they continue to demonstrate the presence of single electron acceptor centers. This indicates that in such cases the Lewis-type acid centers are available for perylene molecules, but no longer available for the thymol blue indicator H_o_ ≤  + 2.8. This is probably the result of the differences in the structure of these indicators [26] (Fig. 1).

Perylene is a flat arrangements of four aromatic rings, whereas thymol blue constitutes a three aromatic ring system, however numerous substituents enhance the surface area of the entire molecule making it uneven.

In both cases, the greatest quantity is encountered when the gels contain 40 and 50% TiO_2_. This indicates that the surface acid centers on investigated gels are of Lewis acid character, located on the surface Ti [IV] ions.

The conducted IR examination revealed the presence of the 3748 cm^−1^ band characteristic for the isolated silanol group ≡Si–OH [13, 16].

### Surface complexes of rhodium applied to TiO_2_-SiO_2_ gels

In aqueous NaOH solutions, RhCl_3_ is converted to yellow trihydrate trihydroxy rhodium [III] Rh (OH)_3_ 3H_2_O (reaction 1):

1 $${\text{RhCl}}_{{3}} \cdot{\text{3H}}_{{2}} {\text{O}}\, + \,{\text{3 NaOH}} \to {\text{Rh }}\left( {{\text{OH}}} \right)_{{3}} \, + \,{\text{3H}}_{{2}} {\text{O}}\, + \,{\text{3 NaCl}}$$

Formally, it constitutes a typical reaction of hard acids and bases [27]: sodium cation and chloride anion as well as soft acids and bases; Rh cation [III] and hydroxyl anion. This reaction runs successfully in the presence of inorganic oxides, as the hydroxide is unstable and its interaction with the surface groups of these oxides stabilizes the hydroxide [28]. The enthalpy of this interaction is negative (ΔH_3_ (kJ mol^−1^) for the following mixed oxides: Ca_2_Nb_3_O_10_ (−35), KNb_6_O_17_ (−37), Rb0.TaO_3_ (−32), and for γ-Al_2_O_3_ and SiO_2_ these enthalpies have positive values of 55 and 25, respectively. Negative enthalpies indicate fairly strong Rh (OH)_3_ carriers. However, this hydroxide deposited on aluminum oxide is stable to the degree allowing to be used as a catalyst in the transformation of aldehydes to primary amides reaction [29].

Another way to obtain trihydroxy rhodium deposited on the surface of inorganic carriers involves depositing of RhCl_3_ on a given carrier in water and then exposing it to UV light [21, 30].

If the driving force in the production of Rh(OH)_3_ from RhCl_3_ in the presence of weak bases is the formation of NaCl, then the production of Rh(OH)_3_ from RhCl_3_ in water in UV light is taking place due to the energy contributed to RhCl_3_ by this radiation (reaction 2):

2 $${\text{RhCl}}_{{3}} \, + \,{\text{3 H}}_{{2}} {\text{O}}\, + \,{\text{h}}\gamma \to {\text{Rh }}\left( {{\text{OH}}} \right)_{{3}} \, + \,{\text{3 HCl}}$$

These hydroxides were formed on 60–100TiO_2_ carriers, as the presence of Rh [III] ions was detected on them by ESCA method (Table 4).

**Table 4:** Degree of oxidation of rhodium ions on the catalyst used

TiO_2_/SiO_2_	mol%	0/100	10/90	20/80	30/70	40/60	50/50	60/40	70/30	80/20	90/10	100/0
Energy.Rh3d_5/2_	eV	307.0	307.7	308.2	308.3	308.6	308.4	309.0	309.0	309/0	309.1	309.2
Valence Rh		Rh[I]	Rh[I]	Rh[I]	Rh[I]	Rh[I]	Rh[I]	Rh[I,III]	Rh[I,III]	Rh[I,III]	Rh,[I,III]	Rh[III]

The HCl yielded in reaction 2, under the conditions of the photocatalyst preparation, cannot become adsorbed on their surfaces due to strongly adsorbed water, and additionally, hydrogen chloride is vigorously complexed with water molecules, thus hindering a possible reversible reaction [31]. Washing photocatalysts obtained with water removes resulting HCl effectively.

However, the ESCA method enabled to detect the presence of rhodium [I] compounds on 0–50% TiO_2_ carriers and on 60–90% Ti carriers next to Rh [III] ions.

The syntheses of rhodium [I]: RhCl (PPh3) 3 and Rh2Cl2 (C2H4) 4 complexes, described in the literature [32, 33] were carried out in excess of phosphine or olefin. In the products, in addition to the rhodium [I] complexes, HCl and oxidized forms of phosphine or olefin as well as water (reactions 3 and 4) were found in reaction products.

3 $${\text{RhCl}}_{{3}} \left( {{\text{H}}_{{2}} {\text{O}}} \right)_{{3}} \, + \,{\text{4 PPh}}_{{3}} \, \to \,{\text{RhCl}}\left( {{\text{PPh}}_{{3}} } \right)_{{3}} \, + \,{\text{OPPh}}_{{3}} \, + \,{\text{2 HCl}}\, + \,{\text{2 H}}_{{2}} {\text{O}}$$

4 $${\text{2 RhCl}}_{{3}} \left( {{\text{H}}_{{2}} {\text{O}}} \right)_{{3}} \, + \,{\text{6 C}}_{{2}} {\text{H}}_{{4}} \, \to \,{\text{Rh}}_{{2}} {\text{Cl}}_{{2}} \left( {{\text{C}}_{{2}} {\text{H}}_{{4}} } \right)_{{4}} \, + \,{\text{2 CH}}_{{3}} {\text{CHO}}\, + \,{\text{4 HCl}}\, + \,{\text{4 H}}_{{2}} {\text{O}}$$

The water molecule from the co-ordination sphere RhCl_3_ 3H_2_O must be the source of hydrogen and oxygen in this reaction. Two chlorines from two HCl molecules must originate from RhCl_3_ molecule, and two electrons to reduce Rh [III] to Rh [I] from Rh-Cl bonds. These effects should result from the following conversions.

The phosphine or ethylene molecules, as strong complexing agents, displace water molecule from the co-ordination sphere of rhodium [III]. The complexes with the possibility of forming “back donation” effect [34, 35], where two electrons of these ligands are located on the vacant 4d-orbital of the rhodium ion [III], and two electrons fill the 4d-orbital of this ion, shift to the vacant anti-bonding orbital of phosphine or ethylene, are likely to yield such changes in the rhodium ion electron environment that affords a hemolytic degradation of two Rh-Cl bonds while forming two chloride radicals and two electrons already located on the rhodium ion [I]. Two chloride radicals attack another molecule of coordinated water, forming two HCl molecules and atomic oxygen. The oxygen attacks the complexed phosphine to form its oxide OPPh_3_ and converts the complexed olefin to an aldehyde. Both products are no longer characterized by such complexing properties as phosphines and olefins and leave the co-ordination sphere of rhodium ions. These released co-ordination sites are filled by “stoichiometric” phosphines or olefins.

These conversions are specific due to the ligands forming the “back donation” effect, as for instance, H_2_O ligands, which do not bring about such effects, fail do reduce Rh [III] ions to Rh [I] ions.

On the other hand, in RhCl_3_ applied onto investigated gels: SiO_2_ and 10–90% Ti carriers with Ti–O–Si bonds, in an aqueous solution during irradiation with UV rays leading to the trihydroxyrhodium product [III], the Rh ion [III] undergoes reduction to Rh ion [I]. This reaction was running in the absence of organic compounds.

Thus, the reduction of Rh [III] ions needs to run only through the homolytic cleavage of two Rh-OH bonds, yielding Rh [I] ion and two hydroxyl radicals:

5 $${\text{2Rh}}\left( {{\text{OH}}} \right)_{{3}} \, + \,{\text{h}}\nu \to {\text{2 Rh}}\left[ {\text{I}} \right]{\text{OH}}\, + \,{4} \bullet {\text{OH}}$$

For homogeneous homologues of rhodium [I] complex obtained, dimeric [X (cyclooctadiene) rhodium (I)]_2_ complexes, where X = Cl.NH_2_ and OCH_3_, the determined bond lengths and angles between them differed slightly, depending on the X ligands, however were contained in the following ranges: Rh-O ~ 2.1 Å bonds, Rh-Rh bond length = 2.8 ÷ 3.3 Å, and X –Rh –X = 86° ÷ 90° for angles, Rh-H_2_O distance ~ 2.2 Å [36–38].

In the investigated carriers, the titanium oxide phases have octahedral anatase structures, where the Ti–O bond length is ~ 2.0 Å, O–O equatorial =  ~ 2.8 Å [39]. In the silica phases, the length of Si–O bonds amounts to ~ 1.6 Å [40].

These bond and angular lengths should appear on the obtained rhodium [III] and rhodium [I] surface complexes.

Heating of the investigated titanium-silica gels at 823 K aimed at stabilizing the oxide structures of mixed gels and silica gel, titanium oxide.

Under these conditions, the titanium oxide is primarily converted to anatase and to rutile, in lower quantities. In these structures, truncated oxide octahedrons appear on their surface, where four oxygen anions are located at equatorial positions, with in-between detected titanium ion IV]. These octahedrons are interlinked through their edges.

In the silica gel, OH vicinal groups -Si(OH)-O-Si(OH) -, geminal = Si(OH)_2_ and isolated ≡Si–OH [40] will appear on its surface.

In titania-silica gels, where the octahedron titanium oxide - silicon oxide tetrahedron groups are present, these structures are connected by Ti–O–Si bonds.

On tetradra surface, the silica will be bound to truncated octahedrons of titanium oxide and only isolated OH groups bound to silicon ion, oxygen anions of Ti–O–Si bonds and exposed titanium ions [IV] will appear on their surface [16].

The rhodium ions [III] in complex compounds most often adopt the octahedron structures by binding six ligands with π and σ bonds. The electron configuration of 4d-rhodium in these complexes is d^6^. The rhodium ion [I] in complex compounds most often adopt the structures of a flat square by binding four ligands with π i σ bonds. The electron configuration of 4d-rhodium in these complexes is d^8^ [41].

In investigated systems, Rh [III] (OH)_3_ on TiO_2_ phases, these hydroxides must be complexed with three non-bonding electron pairs on the surface oxygen orbitals to form octahedral structure. In the case of Rh [I] OH complex, it must be co-ordinated by three non-bonding electron pairs on the surface oxygen orbitals to form a flat square structure.

**Fig. 2:**
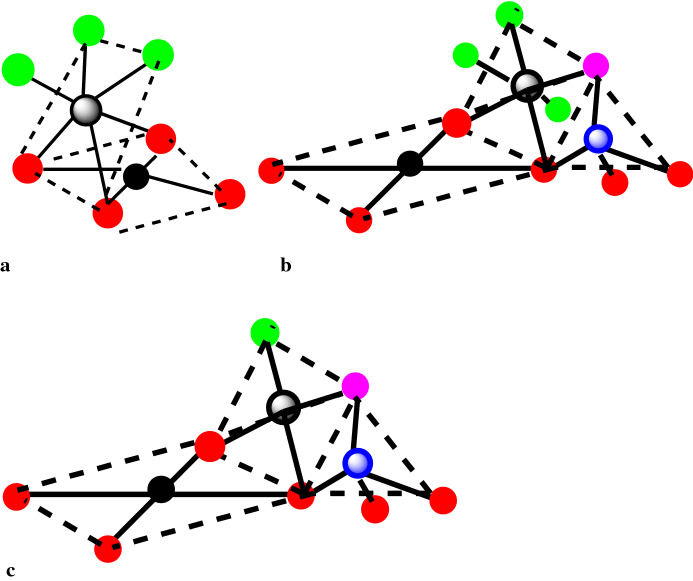
Suggested models: **a** Rh[III](OH)_3_ deposited on anatase crystal; **b** Rh[III](OH)_3_ on the surfaces of titania-silica phases; **c** Rh[I] complex on the surfaces of titania-silica phases. Colours: *red balls* oxygen anions bound with titanium ion; *black balls* titanium ions, *silver balls* rhodium ions, *green balls* hydroxyl anions bound with rhodium ion, *blue ball* silicon ion, *yellow ball* hydroxyl anion bound with rhodium ion, *purple ball* hydroxyl anion bound with silicon ion. *Dotted lines* demonstrate square planes on the surface of anatase and amorphous TiO_2_ as well as in rhodium complexes

These data were employed for proposing the following models of the obtained surface rhodium complexes-Fig. 2.

In the model Rh(OH)_3_/anatase system in the octahedral rhodium complex, two TiO_2_ oxygen anions and two trihydroxy rhodium trihydroxy anions appear in equatorial positions, whereas one TiO_2_ oxygen anion and one rhodium trihydroxy hydroxyl anion in axial positions.

In octahedral rhodium complexes deposited on the surfaces of titania-silica 50% Ti carriers, two oxygen anions of TiO_2_, one oxygen anion of the hydroxyl group bound with silicon ion and one anion of the hydroxyl group of the trihydroxy rhodium appear in equatorial positions. The remaining two hydroxyl groups of this hydroxide are located in axial positions.

The rhodium ion [III] has the d^6^-electron configuration in the complex of octahedron structure and when all electron spins are paired in this system (low spin configuration), the Jahn–Teller effect is taking place. In such a complex, if the ligands are of a donor character, then the lengths of metal-axial ligand bond will be elongated, and such deformation is trigonal. This results in splitting of the e_g_ orbital with two electrons located on the lower d_z_^2^ orbital.

Investigated rhodium [III] trihydroxy surface complexes complexed with three surface donor oxygen of the carrier should be in such situation model b, Fig. 2. In this model, two Rh-OH bonds are in axial positions.

In the model complex of rhodium [I] c-structure, two oxygen donor TiO_2_ ions are in equatorial positions; one donor oxygen anion of the hydroxyl group bound with silicon ion, and one hydroxyl anion.

In rhodium [I] complexes of ML_4_ flat square structure [34, 35, 42], the dz^2^-orbital is characterized by a higher energy than the d_zy_ and d_zx_ orbitals, however the energy is lower than in the case of d_xy_ orbital, whereas the d x^2^-y^2^ orbital has such a high energy that it hinders electron from being shifted from lower d-orbitals. This facilitates transferring the electrons from lower orbitals to adequately low energy anti-bonding ligand orbitals, whilst maintaining orbital symmetry. It is favored by donor ligands.

Such a situation should take place in the resulting surface rhodium hydroxyl complexes [I].

In all model rhodium complexes, hydroxyl groups are bonded with rhodium ions by σ-bonds, whereas the remaining bonds are coordinating ones.

In the case of the Rh (OH)_3_/anatase system, the equatorial Rh-O bonds and Rh-O axial bonds, due to the tight crystal lattice of the carrier, will have difficulties in elongation, as ensured by Jahn–Teller effect [33–35].

Each two hydroxyl groups in this structure are located in *cis* positions. Such a grouping in the ruthenium pincer complexes, according to D. Milstein [43], is a transition complex towards this catalyst, in the photolytic water splitting reaction into hydrogen and oxygen. Such system is also mentioned in the theoretical considerations by W. E. Piers [44] on the reaction towards transition metals.

Taking into the account the previously mentioned lengths of bond and angles in the olefinic complexes of rhodium [I] as well as the proposed structures of investigated rhodium [III] and rhodium [I] surface complexes: a, b, and c; the specific surface areas of the carriers and the Avogadro number, it was possible to perform calculation of surface areas occupied by examined complexes on relevant carriers.

The calculated surface areas of rhodium [III] complexes amount to ~ 30 Å^2^: for two truncated octahedrons on TiO_2_.

For truncated TiO_2_ and SiO_2_ tetrahedron, the surface area of ​​rhodium [III] complexes amounts to ~ 25 Å2.

The amount of RhCl_3_ applied per gram of carrier = 6 10^–6^ mol/g and on the selected specific surface areas of the carrier was used for calculating the surface concentrations of rhodium complexes. For the Rh50% TiO_2_, this concentration amounted to 0.14 mol%, for SiO_2_–0.09 mol%, whereas for TiO_2_ = 2 mol%.

This indicates that the carrier surface constitutes the dominant area of the photocatalysts obtained and its properties must add to the properties of rhodium centers.

### Water photolysis

The study of photolytic water splitting reaction against the examined photocatalysis yielded the following results, as presented in Table 5.

**Table 5:** Quantity of the hydrogen against the Rh/TiO_2_–SiO_2_ photocatalysts obtained

TiO_2_ content	mol% TiO_2_	0	10	20	30	40	50*	60	70	80	90	100**
Hydrogen amount	cm^3^ H_2_/g_cat_	0.02	0.4	0.6	0.4	4.6	5.4	1.3	1.0	0.8	0.2	0.02

*0.02 cm^3^/g_cat_ of hydrogen against the 50 mol% TiO_2_ – 50 mol%_._SiO_2_ carrier examined

The obtained quantity of hydrogen in the examined reactions demonstrate that the rhodium [I] complexes deposited on the surface of amorphous silica gel exhibit a relatively poor activity.

The rhodium [III] complexes deposited on the anatase surface exhibit a comparable low activity. The SiO_2_ carrier itself demonstrated no activity, whereas the anatase – only a slight amounts.

In the carriers containing from 10 to 30 mol% TiO_2_, the dominant phase belongs to amorphous silica gel, which barely activates rhodium complexes, however a slight increase in hydrogen yield is attributable to the increasing number of Ti–O–Si bonds.

In turn, in the 40–50% TiO_2_ carriers, the titanium-silica phase is dominant, which is made up of TiO_2_ octahedrons and SiO_2_ tetrahedra linked by Ti–O–Si bridges. These systems activate rhodium [I] complexes fairly strongly, which are deposited on these carriers, on the 50% TiO_2_ carrier in particular.

In the carriers containing 60–90% TiO_2_, increasing quantites of anatase crystals inactivating the rhodium [III] complexes are introduced to the titanium silica phase, however the remaining surface areas of the titanium-silica phases ensure a relatively high activity of investigated photocatalysts, although they decrease with the increasing amount of TiO_2_ in the carriers.

As mentioned earlier, the rhodium [I] complexes occupy a tiny part of the carrier surface areas and the possible catalytic properties of a given carrier may add to the activity of the rhodium centers. To verify this, water splitting studies were carried out in standard conditions in the presence of the 50% TiO_2_ carrier, the most effective photocatalytic system. We obtained 0.20 cm^3^ of hydrogen.

Slightly higher yields (0.028 cm^3^/g cat., 24 h) were obtained by testing the photolytic water splitting reaction on mixed titania-silica gels.

Thus, in the case of the most effective photocatalyst tested, 5.38 cm^3^ of water converted on rhodium [I] complexes, and 0.02 cm^3^ - on the centers on the carrier surface 50% of TiO_2_. This indicates that rhodium centers are 270 times more active.

The oxygen vacancies feature the centers of water photolysis on TiO_2_ and mixed titania-silica gels [14, 45]. They result from the release of atomic oxygen from the surface bridges: Ti–O–Ti and Ti–O–Si when exposed to UV radiation Reactions 6–9.

6 $${\text{Ti}}{-}{\text{O}} - {\text{Ti}}\, + \,{\text{h}}\nu \to {\text{Ti}}^{{{3} + }} \cdot {\text{Ti}}^{{{3} + }} \, + \,{\text{O}}$$

7 $${\text{Ti}}^{{{3} + }} \cdot {\text{Ti}}^{{{3} + }} \, + \,{\text{H}}_{{2}} {\text{O}} \to {\text{H}}_{{2}} \, + \,{\text{Ti}}{-}{\text{O}} - {\text{Ti}}$$

Summy: $${\text{TiO}}_{{2}} \, + \,{\text{h}}\nu \to {\text{H}}_{{2}} \, + \,{\text{O}}$$

8 $${\text{Ti}}{-}{\text{O}} - {\text{Si}}\, + \,{\text{h}}\nu \to {\text{Ti}}^{{{2} + }} \cdot {\text{Si}}^{{{4} + }}\, + \,{\text{O}}$$

9 $${\text{Ti}}^{{{2} + }} \cdot {\text{Si}}\, + \,{\text{H}}_{{2}} {\text{O}} \to {\text{Ti}}{-}{\text{O}} - {\text{Si}}\, + \,{\text{H}}_{{2}}$$

· - oxygen vacancy.

The release of atomic oxygen from Ti–O–Ti bonds leads to the formation of two Ti^3+^ cations, separated by oxygen vacancy in the reactions of homolytic cleavage of Ti–O bonds. Such system aims to restoring the initial, stable surface structure of TiO_2_, and in reaction with water, it reconstructs the oxygen bridge whilst liberating a hydrogen molecule -H_2_.

In the case of titania-silica carriers in the Ti–O–Si bridge, only the Ti^4+^ cation is able to take in the electrons resulting from the homolytic splitting of the Ti–O and Si–O bonds and the oxygen vacancy has adjacent Ti^2 +^ and Si^4 +^ cations.

Ti^2+^ cations represent stronger reducing agents than Ti^3+^ cations and they release hydrogen from the water molecule more effectively. Therefore, titania-silica gels are more active in this reaction.

**Fig. 3:**
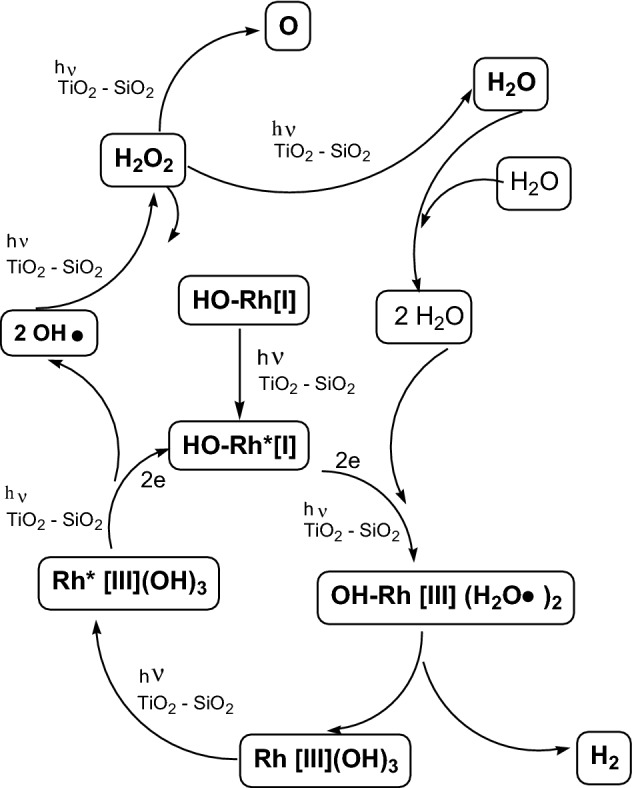
Proposed mechanism of the water photospling reactin on studied rhosium catalysts

The above described data on the acidity of investigated titania-silica gels and the proposed surface structure of rhodium complexes: a, b and c - Fig. 2 were used for proposing the course of the photolytic water splitting into a hydrogen molecule -H_2_ and atomic oxygen -O, in the presence of a photocatalyst containing 50% TiO_2_ (Fig. 3):

The rhodium ion [I] in the complex was linked by a σ-bond with the hydroxyl group and by co-ordination bonds with three surface oxygen anions of the carrier. In the presented rhodium complexes in Fig. 3, the coordination ligands were removed and appear as: HO-Rh for the sake of simplicity.

The previously presented activity of the carriers applied on rhodium complexes ensured their stability. The complexes must be properly activated in order to enter the photocatalytic cycle of water splitting.

In transition metal complexes, the energy in the UV waverange causes electrons to travel from the highest-occupied molecular orbitals in the transition metal ions to the lowest orbitals with no electrons, thus producing excited ions.

In the Wilkinson-type catalysts, such as Rh [I] complexes, as reported earlier, the cleavage of one Rh-O-carrier bond must take place to ensure two vacant orbitals for the formation of the Rh [III] octahedron.

The electrons located on the s- and p-atomic orbitals of metal ions and ligands form molecular orbitals with the lowest energy values for their complexes, while those originating from p atomic orbitals are characterized by a higher energy. Molecular orbitals of nd-electrons have even higher energy and are identical to the d-atomic orbitals of a specific metal ion. Each of these orbitals can split into orbitals of different energy value depending on the structure of the complex, the type of ligands and the energy absorbed [41].

Thus, these two effects: the interaction of the carriers used and the UV radiation of λ ≥ 370 nm, produced appropriately activated rhodium ions, which initiated the water splitting reaction run and ensured the subsequent stages of this reaction.

The resulting activated rhodium complexes have been presented in the diagram as HO-Rh *[I] and HO-Rh *[III].

The whole cycle of the investigated photolytic water reaction consists of two stages. In the first “hydrogen” step, in total hydrogen and two hydroxyl radicals are released from two water molecules complexed with rhodium ions. In the other “oxygen” step, atomic oxygen and water are obtained from two hydroxyl radicals via the obtained hydrogen peroxide.

The first stage of photolytic water splitting reaction starts with the conversion of the surface complex of rhodium [I] to rhodium [III] and is of an oxidizing addition character.

It consists of the following sub-steps;

– The Rh*[I] complex incorporates two water molecules with no effect on the level of oxidation of rhodium ion;

– Two electrons from the 4d-molecular orbital of the rhodium ion [I]: b_1g(x2-y2)_) are transferred onto antibonding molecular orbitals: 2b_2_*, two complexed water molecules, this is facilitated by oxygen donor ligands of the carrier, rhodium [III] being already a rhodium ion.

– The interorbital reaction of electrons with protons in the OH groups of complexed H_2_O molecules, yielding two hydrogen atoms and two OH^−^ groups:

10 $${\text{2e}}^{ - } \, + \,{\text{2 H}}{-}{\text{O}} - {\text{H}} \to {\text{2 H}}\, + \,{\text{2 HO}}^{ - }$$

– Dimerization of two hydrogen atoms to H_2_:

11 $${\text{2H}} \to {\text{H2}}$$

– Two OH^−^ groups become incorporated to Rh [III] already formed.

These intermediate stages are related to the reconfiguration of molecular orbitals of rhodium and water molecules.

**Fig. 4:**
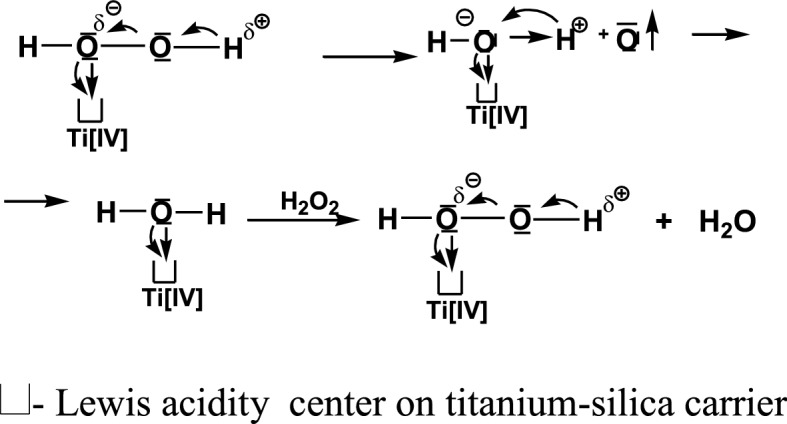
Proposed mechanism

In the case of the rhodium complex [I] studied, which is a flat square, the molecular orbital diagram for such complexes is as follows [41]:


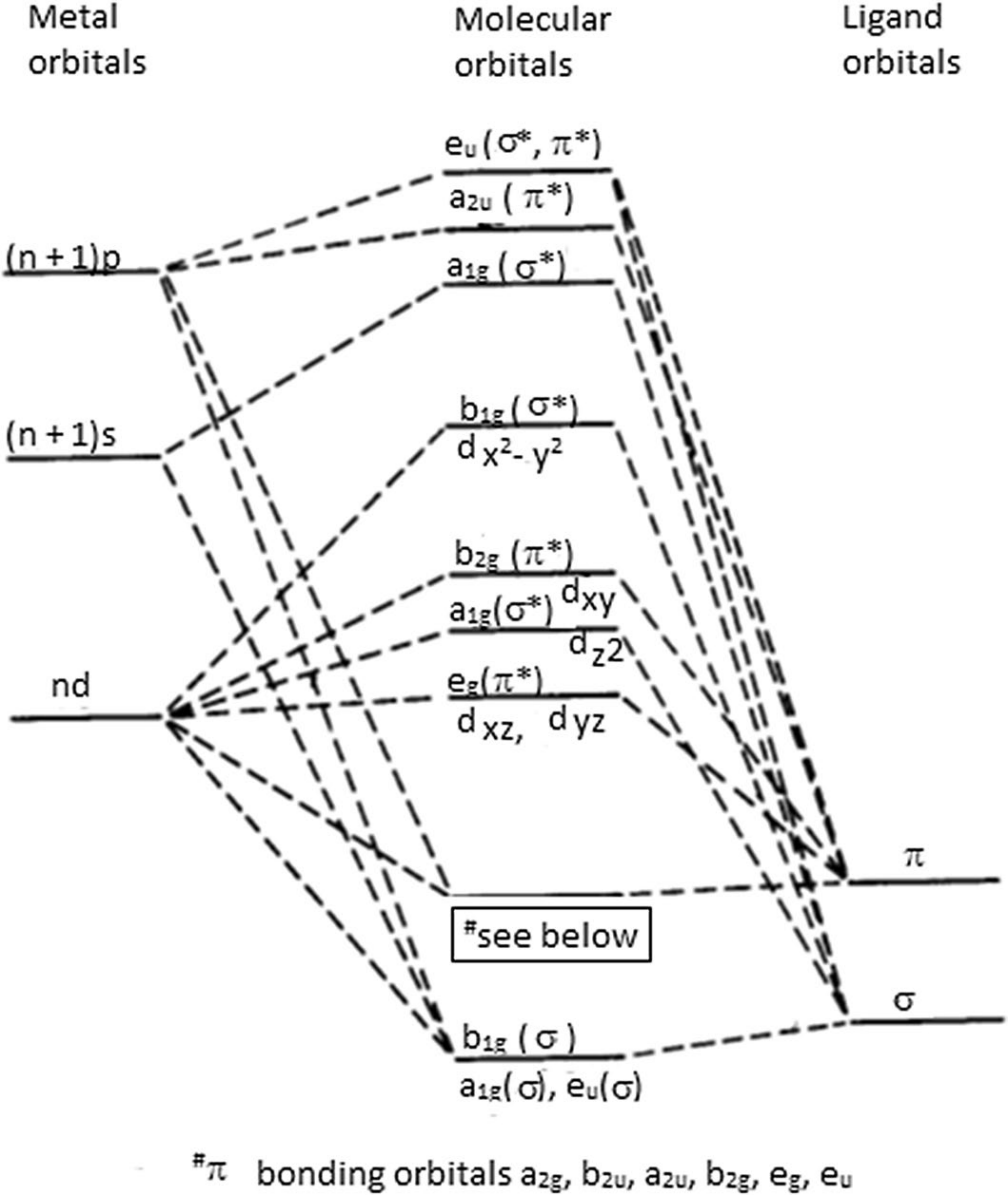
The bonding atomic (n + 1)s metal orbitals and σ- ligand bonds form the lowest orbitals having a comparable energy, while the molecular orbitals from the binding (n + 1)p metal orbitals (n + 1)p and π ligands also have the same energy, however they are already granted the increased energy (Fig. 4).

Molecular nd-orbitals appear even higher; they are identical to metal nd-orbital and their mutual position is typical for flat square structure metal complexes. Antibonding orbitals of metal and ligand specific orbitals are located on upper position.

Electron d-orbitals configuration and their mutual energy values of the investigated surface L_4_Rh [I] complex, where rhodium ion is bound to three surface oxygen ligands (π-bonding) and to one hydroxyl group (σ-bonding):

L_4_Rh [I]: 4d^8^ d_zx_^2^ = d_zy_^2^ < d_z2_^2^ < d_xy_^2^ ≪ d_x2 – y2_^o^ (components of higher MO orbitals).

5s^2^ 6p^6^ (component of lower MO orbitals).

$$\sigma {\text{OH}}\,{3}\pi {\text{O}}$$

L = σ-ligands OH or π O surface ligands.

Electron configuration L_4_Rh [I] + exposition to UV light – transfer of 2 elektrons from d_xy_ onto d _x2- y2_ [34, 35, 42]:

L_4_Rh [I] + UV: 4d^8^-d_zx_^2^ = d_zy_^2^ < d_z2_^2^ < d_xy_^o^ ≪d_x2 – y2_^2^.

$${\text{5s}}^{{2}} {\text{6p}}^{{6}}$$

$$\sigma {\text{OH}}\,{3}\pi {\text{O}}$$

Electron configuration L_4_Rh [I] + UV* and deletion of oxygen carrier ligand.

$$*{\text{L}}_{{4}} - {\text{L}} \to {\text{L}}_{{3}}$$

$${\text{L}}_{{2}} {\text{Rh}}*\left[ {\text{I}} \right]\, + \,{\text{UV - L}}:{\text{ 4d}}^{{8}} {\text{ - d}}_{{{\text{zx}}}}^{{2}} \, = \,{\text{d}}_{{{\text{zy}}}}^{{2}} \, < \,{\text{d}}_{{{\text{z2}}}}^{{2}} \, < \,{\text{d}}_{{{\text{xy}}}}^{{\text{o}}} \, \ll \,{\text{d}}_{{{\text{x2}}{-}{\text{y2}}}}^{{2}}$$

$${\text{5s}}^{{2}} {\text{6p}}^{{4}} {6}_{{\text{p}}}^{{\text{o}}}$$

$$\sigma {\text{OH}}\,2\pi {\text{O}}$$

Bonding of H_2_O to L_3_Rh [I] with no effect on rhodium ion oxidation.

$${\text{L}}_{{2}} {\text{Rh}}*\left[ {\text{I}} \right]\, + \,{\text{UV}}:{\text{ 4d}}^{{8}} {\text{ - d}}_{{{\text{zx}}}}^{{2}} \, = \,{\text{d}}_{{{\text{zy}}}}^{{2}} \, < \,{\text{d}}_{{{\text{z2}}}}^{{2}} \, < \,{\text{d}}_{{{\text{xy}}}}^{{2}} \, \ll \,{\text{d}}_{{{\text{x2}}{-}{\text{y2}}}}^{{2}}$$

$${\text{H}}_{{2}} {\text{O}}$$

$${\text{5s}}^{{2}} {\text{6p}}^{{4}} {6}_{{\text{p}}}^{{2}}$$

$$\sigma {\text{OH}}\,{2}\pi {\text{O H}}_{{2}} {\text{O}}$$

Transfer of two electrons from rhodium ion [I] on two water particles Rh [III] L_3_Rh*[III] L_3_Rh*[III] + UV: 4d^8^-d_zx_^2^ = d_zy_^2^ < d_z2_^2^ < d_xy_^2^ ≪ d_x2 – y2_^2+^

$${\text{H}}_{{2}} {\text{O}} \bullet$$

$${\text{5s}}^{{2}} {\text{6p}}^{{4}} {6}_{{\text{p}}}^{{2}} {\text{MO b}}_{{{\text{2u}}}}$$

$$\sigma {\text{OH 2}}\pi {\text{O H}}_{{2}} {\text{O}} \bullet$$

Formation of octahedral complex L_3_Rh [III] (OH)_3_ + H_2_, [].

L_3_Rh*[III] + UV: 4d^8^-d_zx_^2^ = d_zy_^2^ < d_z2_^2^ < d_xy_^2^ ≪ d_x2 – y2_^2+^

$${\text{OH}}^{ - }$$

$${\text{5s}}^{{2}} {\text{6p}}^{{4}} {6}^{{2}} \, + \,{\text{H}}_{{2}}$$

$$\sigma {\text{OH 2}}\pi {\text{O OH}}^{ - }$$

The formation of the final octahedral L_3_Rh[III](OH)_3_ complex through conversion in the co-ordination sphere of the Rh[III] complex: transfer of the OH^−^ group from the 6p-orbital to the vacant 4 d_x2-y2_^2+^ orbital, resulting in a new occupation of this orbital 4 d_x2-y2_^1+^(OH) and the restoration of the 6p^2^ –O _oxygen ligand carrier_, transfer of the electron from 4d_xy_^2^(OH^−^) orbital to the 4 d_x2-y2_^1+^(OH) orbital yielding 4 d_x2-y2_^2^ (OH) and 4d_xy_^2^(OH), rearrangement of the orbitals position in terms of energy from d_z2_^2^ < d_xy_^2^ (OH) to d_z_^2^(OH) > d_xy_^2^, due to occupation requirements of d-orbitals d in the octahedron structure:

L_3_Rh*[III]: 4d^8^- d_zx_^2^ -d_xy_^2^- d_zy_^2^ < d_z2_^2^- d_x2 – y2_^2^.

$$\sigma {\text{OH }}\sigma {\text{OH}}$$

$${\text{5s}}^{{2}} {\text{6p}}^{{6}}$$

$$\sigma {\text{OH 3}}\pi {\text{O}}$$

Thus, the resulting rhodium [III] complex has a typical octahedral structure where d_z2_^2^ and d_x2 – y2_^2^ orbitals occupy axial positions, whilst the lower energy orbitals: d_zx_^2^-d_xy_^2^-d_zy_^2^ occupy equatorial positions.

It should be noted that this stage is formally corresponding to the reaction of water with metals of redox potential above zero. The example of such a reaction is the reaction of metallic sodium with water leading to the release of hydrogen and yielding appropriate hydroxide. In order to achieve the structure of neon, the sodium atom releases an electron, which needs to shift to water orbitals and evoke conversions in this reaction, as presented in reactions 10 and 11.

As regards the study of organometallic complexes with hydroxyl ligands, Piers W. [44] noted that if a metal ion in the M^n^ oxidation state bound two water molecules while releasing a hydrogen molecule to form a complex with the M^2+^(OH)_2_ -group (oxidative addition stage), then in the reductive elimination hydrogen peroxide –H_2_O_2_ would be produced when exposed to UV light, with the restoration of the original complex with the –M^n^ ion.

In our case, there are three hydroxyl groups in the trihydroxy rhodium [III] obtained in the oxidative addition step, and hence these two can take part in the postulated reductive elimination.

The formation of hydrogen peroxide in the investigated reaction should take place through the following routes. The Rh (OH)_3_ obtained is a stable molecule and Rh [III] ion must be activated to the Rh*[III] ion for catalytic conversion. It occurs immediately, as the catalytic system is continuously exposed to UV radiation. The transfer of electrons to Rh^*^[III] cation from two hydroxyl ligands formed in the oxidative addition step needs to take place through the known homolytic cleavage of σ- metal–ligand [] bonds, i.e. homolytic cleavage of two Rh*(OH) bonds resulting in the initial rhodium complex [I] and 2 hydroxyl radicals, in the examined case—reaction 12.12

This process should be applied to hydroxyl groups in the axial positions of d_z2_ and 4d_x2 – y2_ orbitals, because, as previously described, octahedral complexes of transition metal with d^6^ electron configuration are subjected to the Jahn–Teller effect, i.e. elongation of the bonds the metal axial ligand and shortening of the bonds the metal-equatorial ligand bonds. This effect is facilitated by donor ligands as well as the energy contributed by UV radiation.

After the departure of hydroxyl radicals on 4 d_z2_ and 4d_x2 – y2_ orbitals, one electron will remain i.e. 4 d_z2_^1^ and 4d_x2 – y2_^1^ and, for the final energy system of the orbitals to assume a plane square configuration, the electron from 4d_x2 – y2_^1^ orbital will transfer to 4 d_z2_ orbital, which will assume 4 d_z2_^2^ electron configuration and will lose the energy by positioning itself below the 4d_xy_ orbital whose energy will increase, however the energy of vacant orbital 4d_x2 – y2_^o^ will increase even more.

These processes will be supported by donor ligands and UV radiation.

This stage completes the “hydrogen” cycle with the interaction of rhodium ions that create Rh[I] Rh[III] Rh[I] cycle, where hydrogen molecule = H_2_ was released from two molecules of complexed water on the rhodium ion [I], and at the same time this step constitutes the beginning of “oxygen” cycle, where atomic oxygen = O and water returning to the cycle, will be released from two hydroxyl radicals.

The cycle runs, as follows: the first step invovles the dimerization of hydroxyl radicals to hydrogen peroxide, reaction 13:

13 $${2} \bullet {\text{OH}}\, + \,{\text{h}}\nu \to {\text{H}}_{{2}} {\text{O}}_{{2}}$$

The rate constant of this reaction during exposure to UV light is 5.2 × 10^9^ 1/Ms, where the rate constant of the reverse reaction:

$${\text{H}}_{{2}} {\text{O}}_{{2}} \, + \,{\text{h}}\nu \to {2} \bullet {\text{OH}}$$

amounts to 3.07 × 10^–5^ 1/Ms [46].

These data demonstrate that the formation of hydroxyl radicals in reaction 12 swiftly leads to the formation of hydrogen peroxide, which, in examined case, quickly degenerates to atomic oxygen and water on the surface of the 50% Ti photocatalyst, where the surface of the free titanium silica phase covers 99% second study reaction 14.

14 $${\text{H}}_{{2}} {\text{O}}_{{2}} \to {\text{H}}_{{2}} {\text{O}}\, + \,{\text{O}}$$

Such a process has also been described by S. Imamura et al. [15], as reported earlier, for titanium-silica gel of the same composition. The activity of these gels has been attributed to the presence of acid centers and Ti–O–Si bonds.

The carriers of tested photocatalysts, also with the same quantity of TiO_2_, exhibit the highest acidity, whereas the rhodium [I] complexes deposited on this carrier turn the most effective in the photolytic water splitting reaction. These properties are also linked to the presence of the Ti–O–Si grouping. As discussed previously, Ti [IV] ion exhibits a relatively strong Lewis acidity in that grouping.

The “oxygen” cycle:

$${2} \bullet {\text{OH}} \to {\text{H}}_{{2}} {\text{O}}_{{2}} \to {\text{H}}_{{2}} {\text{O}}\, + \,{\text{O}}$$

This is irreversible, as its end products involve a thermodynamically stable water molecule and atomic oxygen which, after dimerization to O_2_, leaves the reaction as a gas.

Hydrogen peroxide exhibits Brønsted acidity [47], which means that one of the O–H bonds is polarized, so that a partial positive charge is found on the hydrogen, while a partial negative charge is located on the titanium ion of the other OH group. Under favourable conditions, this leads to the dissociation of hydrogen as a proton.

The role of oxygen being released from the ionized peroxide molecule in the presence of titanium silica gel can be presented as follows: Diagram 2.

The ionized hydrogen peroxide molecule becomes complexed on the surface Ti [IV] ion having the said Lewis acidity, through the oxygen ion with a partial negative charge. This entails further ionization of the peroxide, yielding a proton -H^+^, while the electrons obtained by the adjacent oxygen from O–H bond release it as the atomic oxygen. At this stage, the complexed oxygen with the acid center of the carrier affords a negative charge which forms a water molecule by binding a liberated proton. Water is complexed by an acid center, however a partially ionized hydrogen peroxide molecule will push it away from this center. This launches another cycle of releasing atomic oxygen and water, which returns to the entire water splitting cycle.

Thus, this pathway starting from hydroxyl radicals to atomic oxygen and water, and requiring the specific conditions as described, adds to the stoichiometry of the photolytic water splitting reaction on the catalysts under study.

If the “hydrogen” cycle yields hydrogen molecule- H_2_, then the “hydroxyl radical conversion” cycle leads to atomic oxygen. In order to maintain a constant standard balance of water splitting reaction, both cycles must run at the same speed.

The obtained findings and literature data allow to formulate the following conclusions:

Initiation of the run of both cycles needs the activation of stable rhodium [I] and rhodium [III] complexes through the interaction of 40–50 mol% TiO_2_ carriers and UV radiation with a wavelength of ≥ 370 nm.

Furthermore, both cycles require the presence of 40–50 mol% TiO_2_ carriers the “hydrogen” cycle as the carrier of the rhodium complexes, the “oxygen” cycle - as the catalyst for the degradation of hydrogen peroxide. These carriers contain the highest number of Ti–O–Si bonds linking TiO_2_ octahedras with SiO_2_ tetrahedra;

Lewis acid centers of H_o_ strength, appearing only on the catalysts containing 40–50 mol% TiO_2_ ensure effective and selective degradation of hydrogen peroxide into oxygen and water.

## Conclusions

– Titanium-silica gel obtained, containing 40–50 mol% TiO_2_ is the most active carrier of rhodium complexes in the investigated photolytic water splitting reaction;

– These gels exhibit the highest acidity determined by H_o_ coefficient;

– Photolytic splitting of RhCl_3_ applied to investigated gels using UV light of λ ≥ 370 nm, has led to the formation of rhodium hydroxy complexes of various degrees of oxidation: Rh [I] complexes appeared with SiO_2_ and gels 10–90 mol% TiO_2_ as carriers, while Rh [III] complexes were formed on gels of 60–100 mol% TiO_2_;

– Rhodium complexes occupy only ≤ 0.02% of the surface of titania-silica carriers;

– The photolytic water splitting reaction, according to our proposal, consisted of two cycles: in the first “hydrogen” cycle, running only on rhodium complexes, in line with a typical mechanism of oxidative addition and reductive elimination, a hydrogen molecule and two OH radicals are released and the initial active rhodium [I] complex is recreated. In the second cycle, the hydroxyl radicals become converted, in the presence of Lewis acid centers appearing on the surface of 40–50% Ti carriers, into atomic oxygen and water is returning to the circulation;

– Both “hydrogen” and “oxygen” cycles must run at the same rate so as to maintain the stoichiometry of H_2_O → H_2_ + O reaction;

– Photocatalytically active inorganic rhodium complexes and titania-silica gels as well as the aqueous environment of the reaction ensure the absence of organic compounds in the reaction environment and thus evoke the stoichiometric run of the photolytic water splitting reaction to hydrogen and oxygen - no reaction of hydroxyl radicals with organic compounds;

– The presence of two types of rhodium and surface catalytic centers of the carrier was necessary to run photolytic water splitting reaction, as well as the specific addition of energy: interaction on rhodium ions by carriers: 40- and in particular, 50 mol% of TiO_2_, and the energy contributed by UV light of λ ≥ 370 nm.
